# Applying neural network algorithms to ascertain reported experiences of violence in routine mental healthcare records and distributions of reports by diagnosis

**DOI:** 10.3389/fpsyt.2024.1181739

**Published:** 2024-09-10

**Authors:** Ava J. C. Mason, Vishal Bhavsar, Riley Botelle, David Chandran, Lifang Li, Aurelie Mascio, Jyoti Sanyal, Giouliana Kadra-Scalzo, Angus Roberts, Marcus Williams, Robert Stewart

**Affiliations:** ^1^ King’s College London Institute of Psychiatry, Psychology and Neuroscience, De Crespigny Park, London, United Kingdom; ^2^ Biomedical Research Centre, South London and Maudsley National Health Service (NHS) Foundation Trust, London, United Kingdom; ^3^ Sandwell and West Birmingham Hospitals National Health Service (NHS) Trust, West Bromwich, United Kingdom

**Keywords:** natural language processing, victimisation, mental health records, CRIS, violence

## Abstract

**Introduction:**

Experiences of violence are important risk factors for worse outcome in people with mental health conditions; however, they are not routinely collected be mental health services, so their ascertainment depends on extraction from text fields with natural language processing (NLP) algorithms.

**Methods:**

Applying previously developed neural network algorithms to routine mental healthcare records, we sought to describe the distribution of recorded violence victimisation by demographic and diagnostic characteristics. We ascertained recorded violence victimisation from the records of 60,021 patients receiving care from a large south London NHS mental healthcare provider during 2019. Descriptive and regression analyses were conducted to investigate variation by age, sex, ethnic group, and diagnostic category (ICD-10 F chapter sub-headings plus post-traumatic stress disorder (PTSD) as a specific condition).

**Results:**

Patients with a mood disorder (adjusted odds ratio 1.63, 1.55-1.72), personality disorder (4.03, 3.65-4.45), schizophrenia spectrum disorder (1.84, 1.74-1.95) or PTSD (2.36, 2.08-2.69) had a significantly increased likelihood of victimisation compared to those with other mental health diagnoses. Additionally, patients from minority ethnic groups (1.10 (1.02-1.20) for Black, 1.40 (1.31-1.49) for Asian compared to White groups) had significantly higher likelihood of recorded violence victimisation. Males were significantly less likely to have reported recorded violence victimisation (0.44, 0.42-0.45) than females.

**Discussion:**

We thus demonstrate the successful deployment of machine learning based NLP algorithms to ascertain important entities for outcome prediction in mental healthcare. The observed distributions highlight which sex, ethnicity and diagnostic groups had more records of violence victimisation. Further development of these algorithms could usefully capture broader experiences, such as differentiating more efficiently between witnessed, perpetrated and experienced violence and broader violence experiences like emotional abuse.

## Introduction

Interpersonal violence is defined as threatened or actual use of physical force or power against another person, involving one or more perpetrators and victims (1). Violence can be categorised in a variety of ways (e.g., physical, sexual, emotional, domestic) but all cause significant physical and mental morbidity within general populations (2–4). Individuals with a severe mental illness have been found to be significantly more likely to experience domestic, physical, and sexual violence compared to the general population (5–8). Despite this, data on violence (all forms) has been inadequately available from healthcare records. This is partly due to the lack of routine enquiry by professionals at points of clinical contact, and partly because instances of violence are difficult to identify in healthcare data in the absence of specific coding systems (9, 10).

Inconsistencies are also present between different mental health services. For instance, individuals from inpatient settings are more likely to have structured data collected on violent incidents, although the form of data collection also varies depending on the type of violence experienced (11). Electronic healthcare records data could help researchers and clinicians understand the occurrence of interpersonal violence (when disclosed), its risk factors, and the level of treatment and support provided. However, research has focused mainly on recorded incidents within inpatient settings, such as using specific violence definitions to examine the prevalence of recorded experiences of physical assault (12). Because most instances are likely to be recorded as unstructured text data, violence experiences across mental healthcare settings cannot be adequately captured without natural language processing (NLP).

A general challenge for using health records data for research is that the most valuable and granular information is frequently contained in text fields (e.g., routine case notes, clinical correspondence) rather than in pre-structured fields; this includes mentions of violence whether experienced as a victim or perpetrated. NLP has been used increasingly to extract information automatically from unstructured text in electronic health records, particularly in mental healthcare, on clinical entities such as diagnosis, symptoms, and treatment (11–14). However, few of these studies have applied NLP to investigate mentions of violence across different clinical samples. One study using NLP reported greater odds of physical victimisation within groups who had an ICD-10 diagnosis of F2x (schizophrenia, schizotypal and delusional disorder), F6x (disorders of adult personality and behaviour), F7x (mental retardation) and F3x (mood disorders) diagnostic groups vs those with an organic syndrome. However, this was specifically examined within an inpatient setting, where victimisation would be expected to be mentioned more regularly than in outpatient samples (11). Another study using NLP found individuals with victimisation to be most commonly diagnosed with psychotic disorders (20.4%) or mood disorders (16.3%) (12). However, this study specifically investigated physical victimisation, rather than other types of experienced victimisation. From these findings, it could be suggested that physical victimisation may be more prevalent in individuals with a diagnosis of a psychotic or mood disorder.

An NLP approach was previously developed to ascertain violence according to its presence, agent (i.e., patient as perpetrator or victim) and certain subtypes (physical, domestic, sexual) (15). This method provided a potential way of furthering research on how professionals and services respond to violence, as well as provide opportunities for monitoring recorded violence victimisation in different groups (16). For example, one application of these NLP algorithms included their use in a study investigating associations of victimisation with adverse mental healthcare outcomes during the early stages of the COVID-19 pandemic (17). Having run these previously developed algorithms across a large mental healthcare data resource, we sought to describe the distribution of interpersonal violence ascertained in this way across different psychiatric settings and diagnostic groups. The output presented here examined the distribution of any recorded violence victimisation, with secondary analysis examining the distribution of specific victimisation types: physical, domestic, and sexual violence. This was a descriptive study testing victimisation seeking primarily to estimate the prevalence of recorded victimisation using the aforementioned NLP algorithm across a large mental health resource. Therefore, we did not have specific hypotheses relating to which diagnostic groups would have higher prevalence of specific victimisation types. However, it was anticipated from the previous studies mentioned, that physical victimisation may be higher in patients with an ICD-10 diagnosed psychotic or mood disorder (F2x, F3x).

## Materials and methods

The study reported in this paper analysed information about violence extracted from the English language text portions of a de-identified secondary care psychiatric electronic health record (EHR), from the South London and Maudsley NHS Foundation Trust. The text consisted of a mix of document types from several EHR fields, including correspondence between clinicians, event notes written by clinicians in day-to-day clinical care, and discharge summaries (18, 19).

### Extracting violence from mental health records text using NLP algorithms

The method by which violence information was extracted from the text is in routine at the UK’s National Institute for Health Research Maudsley Biomedical Research Centre, where it is regularly run over the dataset. The full method, and its evaluation, has been previously reported (15). We provide a summary here for convenience.

As a first step, a list of violence-related keywords based on literature, clinical experience and informatics expertise was created. Seventeen keywords were assembled in this respect. Next, a technique called sequence classification, a common sub-task in NLP, was implemented. This involves obtaining text sequences that contain one of the listed violence-related key words, and manually labelling them as being indicative or not of five binary classes (a mention of victimisation, perpetration of violence, or general mention (as victim, witness or perpetrator) of domestic violence, physical violence or sexual violence) by multiple annotators. Guidelines were then developed on how to annotate further text sequences based on discussions with these annotators of their experiences (e.g., what text sequences would be more indicative of victimisation vs other text sequences). Inter-annotator agreement was estimated on a subset of the data labelled, giving agreements in this case of 82%-96%, and Cohen’s kappa coefficients of 60%-85% (15). As previously stated, the selection of keywords, labelling guidelines, characteristics of the labelled text and the labelling process are fully described in a previous (open access) publication (15). After measuring inter-annotator agreement on what would be classed as one of the five binary classes, separate binary classification models were trained from the labelled data, one for each of these five classes. Models were built by adapting a widely used transformer model (a type of neural network model), BioBERT (20). BioBERT was adapted using the Hugging Face Bert-For-Sequence-Classification interface (21) adding a single classification layer to the standard transformer model. Cross entropy loss with custom weight parameters was used to account for dataset imbalance. Each model created in this way classifies a text sequence as being a member or not a member of a class, such as physical violence or domestic violence. We refer to these as “instance level” mentions of violence. These instance level text sequences are derived from documents, such as clinician notes. The final algorithm labels any document that contains one or more text sequence instance of a given class with that same class, thus creating a “document level” label. For example, if a document contains two sequences labelled as being in the physical violence class, and three sequences in the domestic violence class, then the document will be labelled as being in the physical violence and domestic violence classes. As documents are written about and linked to patients, we are then able to draw conclusions about those patients. Blind testing of the final NLP algorithms on 1411 random documents gave document level F1 statistics of 0.90, 0.85, 0.98, 0.93, and 0.93 for victimisation, perpetration, physical, domestic, or sexual attributes respectively (15).

### Data resource

As with the NLP development, data for the analyses presented here were extracted from the case register of the South London and Maudsley NHS Foundation Trust (SLaM). SLaM is a large secondary care mental healthcare provider, serving around 1.3 million residents of a defined catchment of four London boroughs (Croydon, Lambeth, Lewisham, and Southwark). SLaM care covers all specialist mental health care, including liaison and crisis teams, community and inpatient services and early intervention services. Electronic health records (EHRs) have been used for all SLaM services since 2006, and the Maudsley Clinical Record Interactive Search (CRIS) platform was established in 2008 in order to retrieve de-identified data from records of patients previously or currently receiving SLaM care (18). The EHR source includes structured fields coding demographic information (e.g., ethnicity, sex, age), and unstructured free text fields from case notes, mental health examinations, personal histories, management plans and correspondence. Within the last decade, a range of NLP algorithms have been developed, whose detailed performance data and descriptions can be found in an open-access catalogue (22). CRIS has a robust, patient-led governance and data security model and has approval as a data resource for secondary analysis (Oxford Research Ethics Committee C, reference 18/SC/0372).

### Analysed sample

For the analyses within this paper, data were extracted for all individuals receiving SLaM services at any point during 2019, defining their demographic and diagnostic status on or as closest as possible to an index date of 1^st^ July 2019 and ascertaining any recorded violence victimisation from the full record up to the end of 2019. The NLP algorithm can assess for a mention of violence victimisation, but it cannot accurately indicate the frequency with which that victimisation has occurred (e.g., three mentions of victimisation in different documents highlighted by the NLP could refer to the same event). As this study is interested in whether individuals have a mention of recorded victimisation in general, patients were classified within two groups based on whether they had one or more mention of recorded violence victimisation in any free text fields occurring within the study period. Records describing the violence victimisation were then further evaluated for the presence or not of physical, domestic, or sexual violence. Because the violence app in its current version does not identify the intersection of violence type and violence victimisation specifically at instance level, performance was re-checked by extracting documents to analyse accordance of each recorded victimisation and type combination. Based on 50 randomly selected positive instances for each, evaluated for the analyses presented in this report, the precision statistics for victimisation for physical violence, domestic violence, and sexual violence were 0.72, 0.72 and 0.62 respectively.

### Measurements

Demographic variables extracted were age, sex, and ethnicity. Age at the index date was categorised and entered in 10-year increments. Ethnicity was categorised into six groups for analysis compiled using census categories (23): ‘Asian’ (Indian, Bangladeshi, Pakistani, Chinese or any other Asian background), ‘Black’ (Caribbean, African or any other black background), ‘White British’ (British), ‘White other’ (Irish or any other white background), ‘Other/mixed’ (White and Asian, White and Black Caribbean, White and Black African, any other ethnic group) and ‘Not stated’. Diagnoses are coded in structured fields in the source record according to the International Classification of Diseases, 10^th^ Edition (ICD-10). Participants were categorised by ICD-10 codes (24) for primary diagnosis (recorded closest to 01.07.2019) as follows: F0x (organic mental disorders), F1x (psychoactive substance use), F2x (schizophrenia, schizotypal and delusional disorders), F3x (mood disorders), F4x (neurotic, stress-related and somatoform disorders), F5x (behaviour syndromes associated with physiological and physical factors), F6x (disorders of adult personality and behaviour), F7x (mental retardation), F8x (disorders of psychological development), F9x (behavioural and emotional disorders with onset during childhood and adolescence), ‘unspecified’ and ‘no axis 1’. In addition, post-traumatic stress disorder (PTSD; F43.1) was ascertained as an individual disorder of interest.

### Statistical analysis

All analysis was conducted in R (version 4.1.2) using various packages (readr (25); dplyr (26); ggplot2 (27);). Descriptive statistics (means, standard deviations, frequencies, and percentages) of age, sex, ethnicity, and victimisation mentions were provided. Patients without any of the sociodemographic data were excluded from analysis. Chi square tests were also conducted to investigate victimisation differences between different demographic groups (age, sex, ethnicity) and diagnostic groups, supplemented by Cramér’s V effect sizes. These results were reported for any recorded violence victimisation, as well as specifically for domestic, physical, and sexual victimisation. Logistic regression analysis was conducted to investigate whether being part of a specific diagnostic group predicted mention of any recorded violence victimisation. Diagnostic groups were defined as separate binary variables for each diagnosis, (e.g., F0x diagnoses vs all other categories). Unadjusted models assessed age, sex, ethnicity, and each binary diagnostic group comparison in relation to presence or not of recorded experiences of recorded violence victimisation. Each of these models (for each sociodemographic variable and separate diagnostic group comparison) was then adjusted for age, sex, and ethnicity. The adjusted models were also conducted within males and female subsamples independently. For secondary analysis, unadjusted and adjusted regressions were conducted to measure whether being part of a specific diagnostic group predicted mention of physical, domestic, and sexual victimisation specifically. Bonferroni correction was used to adjust for multiple comparisons, whereby the alpha value was lowered to account for the number of comparisons performed (0.05 divided by number of tests conducted). P values from the regression analysis were considered significant if they were lower than the adjusted value. Multicollinearity tests using the R function vif() within the [car package] were undertaken to avoid issues with overlapping predictor variables. The predictor of being of age 91-100 was not added to the adjusted regressions, as it was highly correlated with other predictor age groups (with a VIF value above five (28)).

## Results

We present results of the violence prevalence analysis based on information extracted using NLP. A full evaluation of the NLP itself can be found in the previously published paper (15). The cohort comprised 60,021 individuals: 56,482 with a F0-F9 diagnosis, 3527 with an unspecified disorder and 12 with no axis 1 disorder recorded. Of the 56,482 individuals with a F0-F9 diagnosis, there were 27,191 (46.3%) with at least one victimisation mention: 26,038 (46.1%) with a mention of physical violence, 22,396 (39.7%) with domestic violence, and 13,558 (24.0%) with sexual violence. The mean (SD) age of the cohort was 37.6 (20.4) years. Distribution frequencies and Chi squared test results for associations with demographic variables and diagnostic group can be found in **Table 1**. Age, sex, ethnicity, and diagnostic group were all significantly associated with any victimisation, physical, domestic, and sexual victimisation mentions. For age groups, violence prevalence showed an inverted-U-shaped pattern of association with highest proportions in the 41-60y groups for all types. All victimisation types were more commonly recorded in women than men. For ethnicity, the highest prevalence of overall victimisation was within the Black ethnic group (62.3%), which was also observed for recorded physical and sexual violence victimisation specifically, but the highest prevalence of domestic victimisation was in the Other/Mixed group. For diagnostic groups, overall recorded violence victimisation prevalence was highest in patients with schizophrenia and related disorders (F2x) or personality disorders (F6x), the same being observed for physical violence. Recorded domestic and sexual violence victimisation prevalence were highest in those with personality disorder diagnoses. Considering effect sizes, as quantified by Cramér’s V statistic, these were moderate (0.2-0.6) for ethnicity and diagnosis and small (<0.2) for age and sex. Most did not vary substantially by violence category apart from sex which had higher effect sizes for domestic and sexual than physical violence, and ethnicity which was strongest for physical violence.

**Table 1:** Distribution frequencies (N(%)) and chi square test statistics measuring group differences in recorded violence victimization or specific physical, domestic, or sexual victimisation in 2019 for each age category, sex, ethnicity, and diagnostic group.

Predictor variable	All patients	Any victimisation		Physical		Domestic		Sexual	
		N (%)	*X^2^ (V)*	N (%)	*X^2^ (V)*	N (%)	*X^2^ (V)*	N (%)	*X^2^ (V)*
**Age**			2118.6* (0.19)		2023* (0.18)		1603.9* (0.16)		1928.8* (0.18)
0-10 years	3220	981(30.47)		875(27.17)		825(25.62)		241(7.48)	
11-20 years	11457	5289(46.16)		5020(43.81)		4493(39.22)		2154(18.80)	
21-30 years	11039	5115(46.34)		4908(44.46)		4365(39.54)		2754(24.95)	
31-40 years	10232	5265(51.46)		4990(48.77)		4543(44.40)		2871(28.06)	
41-50 years	8225	4600(55.93)		4450(54.10)		3713(45.14)		2526(30.71)	
51-60 years	7317	4249(58.07)		4073(55.66)		3325(45.44)		2231(30.49)	
61-70 years	3456	1654(47.86)		1619(46.85)		1234(35.71)		805(23.29)	
71-80 years	2808	814(28.99)		783(27.88)		596(21.23)		298(10.61)	
81-90 years	2267	440(19.41)		441(19.45)		321(14.16)		86(3.79)	
**Sex**			563.45* (0.10)		397.02* (0.08)		1980.7* (0.18)		1640.1* (0.17)
Female	29823	15567(52.20)		14710(49.32)		14294(47.93)		9036(30.29)	
Male	30198	12840(42.52)		12449(41.22)		9121(30.21)		4930(16.33)	
**Ethnic group**			4097.4* (0.26)		4231.1* (0.27)		2870.3* (0.22)		2058.2* (0.19)
White British	22317	11413(51.14)		10896(48.83)		9570(42.88)		5799(25.98)	
White Other	4586	2302(50.19)		2209(48.17)		1940(42.30)		1108(24.16)	
Black	11433	7120(62.28)		6967(60.94)		5596(48.95)		3706(32.41)	
Asian	2955	1586(53.67)		1536(51.98)		1270(42.98)		699(23.65)	
Other/Mixed	5100	2954(57.92)		2802(54.94)		2574(50.47)		1485(29.12)	
Not Stated	12630	3032(24.01)		2749(21.77)		2456(19.45)		1169(9.26)	
**Diagnostic group**			6182.4* (0.32)		6365.3* (0.33)		4548.6* (0.28)		5168* (0.29)
F0-F09	4287	842(19.65)		839(19.57)		593(13.83)		209(4.88)	
F10-F19	5560	2527(45.45)		2360(42.45)		1938(34.86)		1081(19.44)	
F20-F29	7212	5467(75.81)		5433(75.33)		3980(55.19)		3003(41.64)	
F30-F39	7166	4119(57.48)		3932(54.87)		3737(52.15)		2182(30.45)	
F40-F49	8296	3997(48.18)		3814(45.97)		3486(42.02)		2042(24.61)	
F50-F59	1878	721(38.39)		664(35.36)		666(35.46)		368(19.60)	
F60-F69	2381	1972(82.82)		1909(80.18)		1808(75.93)		1442(60.56)	
F70-F79	787	412(52.35)		423(53.75)		271(34.43)		188(23.89)	
F80-F89	3273	1225(37.43)		1163(35.53)		953(29.12)		388(11.85)	
F90-F98	15642	5909(37.78)		5501(35.17)		4964(31.74)		2655(16.97)	
Unspecified	3527	1211(34.34)		1114(31.59)		1013(28.72)		404(11.45)	
No axis 1	12	5(41.67)		7(58.33)		6(50.00)		4(33.33)	

*All p-values <.01; Cramér’s V effect size provided.

For overall recorded violence victimisation, results from unadjusted and adjusted logistic regression models are displayed in **Table 2**. In adjusted models, the same mid-life peaks in age distribution were observed as in unadjusted analyses, as were associations with female sex and with Black, Asian, and Other/Mixed ethnic groups compared to the White British reference. Additional analysis conducted in males and females separately found few differences between the sex of patients (**Supplementary Table 1**). For diagnoses, when analysed individually against all other diagnostic groups, significantly higher odds of recorded violence victimisation were observed in patients with schizophrenia and related disorders (F2x), affective disorders (F3x), PTSD, and personality disorders (F6x). In secondary analyses of specific violence types, findings were similar for physical and domestic violence (**Supplementary Tables 2, 3**, respectively). Findings for sexual violence differed in that no association was found with Asian ethnic groups compared to the White British reference; they were similar in all other respects (**Supplementary Table 4**).

**Table 2:** Unadjusted and fully adjusted logistic regression models for having at least one record of violence victimisation (any type) in 2019.

	Unadjusted	Fully adjusted
Predictor	OR(95% CI)	OR(95% CI)
Age group		
0-10 years	0.50(0.47-0.55)**	0.43(0.39-0.47)**
11-20 years	0.99(0.47-0.55)	0.81(0.77-0.86)**
21-30 years	Reference group	
31-40 years	1.23(1.16-1.30)**	1.13(1.07-1.20)**
41-50 years	1.47(1.39-1.56)**	1.33(1.25-1.41)**
51-60 years	1.60(1.51-1.70)**	1.36(1.28-1.45)**
61-70 years	1.06(1.51-1.70)	0.88(0.81-0.96)**
71-80 years	0.47(0.43-0.52)**	0.36(0.33-0.40)**
81-90 years	0.28(0.25-0.31)**	0.20(0.18-0.23)**
Sex		
Female	Reference group	
Male	0.68(0.66-0.70)**	0.64(0.62-0.65)**
Ethnic group		
White British (%)	Reference group	
White Other (%)	1.05(0.99-1.12)	1.02(0.95-1.08)
Black (%)	1.72(0.99-1.12)**	1.73(1.65-1.82)**
Asian (%)	1.21(1.12-1.30)**	1.22(1.12-1.32)**
Other/Mixed (%)	1.44(1.35-1.53)**	1.41-1.32-1.50)**
Not Stated (%)	0.33(0.31-0.35)**	0.30(0.28-0.31)**
Diagnostic group		
F0-F09	0.25(0.23-0.27)**	0.32(0.29-0.35)**
F10-F19	0.92(0.87-0.97)**	0.63-0.60-0.67)**
F20-F29	4.08(3.86-4.32)**	3.19(3.00-3.40)**
F30-F39	1.59(1.51-1.67)**	1.42(1.35-1.50)**
F40-F49	1.04(0.99-1.09)	0.95(0.90-1.00)*
PTSD	4.84(4.19-5.62)**	4.21(3.62-4.91)**
F50-F59	0.69(0.62-0.75)**	0.50(0.45-0.55)**
F60-F69	5.69(5.12-6.35)**	4.66(4.17-5.22)**
F70-F79	1.23(1.07-1.41)**	0.87(0.75-1.00)
F80-F89	0.65(0.6-0.70)**	0.80-0.63-0.86)**
F90-F98	0.59(0.56-0.61)**	0.72(0.69-0.75)**
Unspecified	0.79(0.24-2.49)	0.51(0.15-1.67)
No axis 1	0.56(0.52-0.60)**	0.68(0.63-0.74)**

OR, odds ratio; CI, Confidence intervals. *p<.05, **p<.01. Bold: significant results after controlling for multiple comparisons (0.05/28 tests conducted, new p level=0.00179). Diagnostic group effect sizes: odds of victimisation among those with the diagnoses compared to all other patients.

Adjusted models controlled for age, ethnicity, and sex.

## Discussion

To our knowledge, this is the first application of NLP algorithms to characterise recorded violence in a large corpus of mental health electronic health records. Considering distribution, violence was most commonly recorded in mid-life age groups, in women compared to men, in patients from minority ethnic groups compared to White groups, and among people diagnosed with schizophrenia and related disorders, affective disorders, PTSD and personality disorders, compared to those with other diagnoses.

The reported prevalence of violence in individuals with a severe mental illness has varied between 4% to 35% (5),, with prevalence of violence in patients with a general mental disorder being 15.2% (compared to 6.9% in those without) (29). Physical, domestic, and sexual violence were recorded in 46%, 40% and 24% of our sample of individuals with a diagnosis of a F0-F9 disorder. These absolute levels should be viewed cautiously in light of the performance levels of the algorithms, which we intend to develop further to improve characterisation accuracy. In particular, it should be borne in mind that status combinations (i.e., between ‘victimisation’ and each violence type) could only be applied at document level. It was therefore conceivable that the victimisation status applied to a different experience of violence in the same document (e.g., sexual violence might have been recorded as a perpetration event in the same document as physical violence received as victimisation, resulting in a false positive ascertainment for recorded sexual violence victimisation). Sub-optimal precision (positive predictive value) will have resulted in an over-estimation of exposure due to false positive instances, while sub-optimal recall (sensitivity) will have resulted in an under-estimation of exposure due to missed instances. Under-estimation will also clearly result from failure to ascertain or record experiences of violence in the source clinical record. Despite this, the associations with demographic and clinical factors, in the directions anticipated, support the applicability of these algorithms, at least as proxy markers of exposure, for analysis over large datasets, even if the performance levels achieved to date do not yet support their use for individual clinical decision support. Importantly, to our knowledge, there are currently no adequate means for quantifying recorded violence victimisation in mental healthcare records (or clinical records for any specialty), so we feel that the approach here at least represents a step towards more inclusive data capture. Relatively high prevalence of recorded violence is consistent with the 17% prevalence for any victimisation ascertained in case notes from a shorter (3-month) period early in the COVID-19 pandemic, a feature that was found to be prospectively associated with increased risk of acute care, emergency referrals, and mortality (30).

Recorded violence was ascertained most frequently in people with diagnoses of schizophrenia and related disorders, affective disorders, PTSD, and personality disorders. The vulnerability of patients within these diagnostic groups to experiences of interpersonal violence has been strongly supported in previous literature

(31–34). Therefore, our results support the notion of having increased screening (for all victimisation types) and victimisation support for these vulnerable groups. Unexpectedly, patients diagnosed with an organic disorder, substance misuse disorder, stress disorder (excluding PTSD), developmental disorder or a disorder with physiological disturbances had significantly less victimisation mentions than other disorders. Previous research has found at least some of these disorders to be risk factors for victimisation, such as research reporting higher rates of victimisation with a substance use disorder compared to those without (32). However, the observed low effect sizes may suggest that disorders such as schizophrenia should be considered a stronger risk factor. In interpreting these findings, it is important to bear in mind the purpose of the algorithm – namely to ascertain violence that has been clinically recorded. It is possible that the nature of some diagnoses encourages the ascertainment and recording of violence; for example, the diagnosis of PTSD would require identification and recording of an index traumatic event, and diagnoses of affective or personality disorders may prompt (and/or result from) a detailed enquiry as to relevant aetiology. In addition, it is important to bear in mind that longer and/or more intensive clinical contact, accompanied by more extensive health records, will increase the likelihood of events being recorded, something which was not adjusted for in these analyses. Patients with briefer contacts with mental healthcare are likely to have less detailed records, which might account for the lack of association with substance use disorder diagnoses. Of note, it is important to bear in mind that the diagnostic categories used in this analysis are very broad ones. There may well be within-category heterogeneity in associations, particularly within the larger groupings of patients with schizophrenia and related disorders, and mood disorders. Evaluation of more specific diagnostic sub-groups was not attempted in this study, aside from PTSD, and we feel that this would demand more specific investigation within broadly defined clinical groups (e.g., mood disorders) rather than across all mental health service users. However, more granular clinical phenotypes might be better ascertained via recorded symptom profiles than specific diagnostic codes, given the potential variability with which coding is likely to be applied in routine practice.

In relation to sociodemographic factors, patients from most minority ethnic groups had significantly higher risk of recorded violence victimisation compared to White British patients. While patients from minority ethnic groups face more barriers that reduce instances of disclosing victimisation in healthcare settings (35), the findings of higher recorded victimisation in these groups has been consistently highlighted in previous literature (36). Also supporting previous research (37), male patients were at a lower risk of victimisation mentions compared to females; this was consistent within all victimisation types. Future research could helpfully investigate whether incidents of victimisations differ between men and women within different diagnostic groups, to ascertain vulnerability and target further support.

### Strengths and limitations

The study described here has important strengths. Firstly, it provides novel findings on how sociodemographic factors and mental health diagnosis associate with the distribution of recorded violence victimisation within clinical record data. The 12-month time period for assessment allowed victimisation to be assessed across a representative sample of patients receiving secondary mental healthcare services, circumventing seasonal variation of victimisation (38, 39). In addition, 2019 was chosen as a recent time period, but one which preceded the COVID-19 pandemic and consequent disruption to services and, potentially, healthcare records. The large sample size increased the precision for the estimate of prevalence of violence mentions and allowed distributions to be investigated across a wide range of disorders. The development of the NLP victimisation application demonstrated the application of machine learning to unlock a complex but clinically important construct, utilising rich and diverse free-text data from a wide array of clinical professionals and groups (15). This approach helps to automate the measurement of victimisation, increasing the number of cases that can be investigated and providing a method that could be used more routinely to monitor victimisation in patients.

One of the important limitations of the NLP algorithm at its current stage of development is the requirement to combine features at document rather than instance level. This means that the algorithm could be raising documents with mixed experiences, e.g. a document raised by the algorithm as having a positive mention of violence victimisation may also include recorded instances of perpetrated violence. Therefore, prevalence of recorded victimisation should be considered with caution and further development of the NLP algorithm is needed to increase precision and recall. In addition, NLP can only be used to ascertain violence which, if it is recorded at all, is done so using terminology that can be reliably ascertained. This will inevitably underestimate true exposure where this is not enquired about and/or not reported by the patient and/or not recorded by the reviewing clinician (9, 40). Finally, the analyses presented here focused on relatively few characteristics as exposures, and only considered the primary diagnosis of the patient (and, as mentioned, within relatively broad diagnostic groupings), not including the additive effects of comorbid disorders that may strengthen or weaken the risk of victimisation.

Considering future directions, clearly further development is required to construct accurate NLP algorithms to allow combinations of features at instance level, and to differentiate more efficiently between witnessed, perpetrated, and experienced violence, as well as encompassing broader experiences (e.g., including emotional abuse). This would aid in our understanding of the complex relationship between violence and mental health diagnoses. Future research into the clinical benefits of synthesizing previous interpersonal violence experienced by patients could aid in the real time decision making of clinicians, although ethical challenges of using NLP methods in practice need to be considered (15).

## Data Availability

The datasets presented in this article are not readily available due to the terms of Ethics and Information Governance approvals and clinical source of the data, CRIS datasets must remain within the SLaM firewall. All data used from this study can remade accessible on request from ris.administrator@slam.nhs.uk with an appropriate research passport or appropriate SLaM honorary contract. Requests to access the datasets should be directed to ris.administrator@slam.nhs.uk.
