# Supplementary material for: Applying neural network algorithms to ascertain reported experiences of violence in routine mental healthcare records and distributions of reports by diagnosis
Source: Front Psychiatry. 2024 Sep 10;15:1181739. doi: 10.3389/fpsyt.2024.1181739 (PMC11420987; doi:10.3389/fpsyt.2024.1181739)
Supplement: Supplementary file 1 [file DataSheet1.pdf]

Supplementary table 1: Fully adjusted logistic regression models stratified by sex of patients having at least one record of violence victimisation (any type) in 2019, fully controlled for age and ethnicity.

Predictor	Male patients OR(95% CI)	Female patients OR(95% CI)
**Age group**		
0-10 years	**0.47(0.42-0.52)****	**0.36(0.31-0.42)****
11-20 years	0.90(0.42-0.53)**	**0.75(0.70-0.81)****
21-30 years	Reference group	
31-40 years	1.16(1.07-1.27)**	1.10(1.02-1.19)*
41-50 years	**1.31(1.19-1.42)****	**1.38(1.27-1.51)****
51-60 years	**1.41(1.29-1.54)****	**1.34(1.22-1.46)****
61-70 years	0.97(0.86-1.09)**	**0.81(0.72-0.91)****
71-80 years	**0.39(0.33-0.44)****	**0.35(0.31-0.39)****
81-90 years	**0.28(0.24-0.34)****	**0.17(0.14-0.19)****
**Ethnic group**		
White British (%)	Reference group	
White Other (%)	0.98(0.89-1.08)	1.04(0.96-1.16)
Black (%)	**1.80(1.68-1.92)****	**1.65(1.54-1.77)****
Asian (%)	**1.36(1.22-1.51)****	1.06(0.95-1.19)
Other/Mixed (%)	**1.42(1.30-1.55)****	**1.39(1.27-1.52)****
Not Stated (%)	**0.26(0.24-0.28)****	**0.33(0.31-0.36)****
**Diagnostic group**		
F0-F09	**0.37(0.32-0.43)****	**0.28(0.24-0.32)****
F10-F19	**0.52(0.49-0.56)****	0.95(0.85-1.06)
F20-F29	**3.59(3.32-3.89)****	**2.76(2.48-3.04)****
F30-F39	**1.47(1.35-1.61)****	**1.38(1.29-1.48)****
F40-F49	0.97(0.90-1.06)	0.93(0.87-0.99)*
PTSD	**3.39(2.73-4.24)****	**5.03(4.07-6.28)****
F50-F59	**0.34(0.26-0.45)****	**0.53(0.47-0.59)****
F60-F69	**3.98(3.32-4.77)****	**5.10(4.42-5.90)****
F70-F79	1.23(1.07-1.41)	0.74(0.58-0.93)*
F80-F89	**0.84(0.77-0.94)****	**0.66(0.58-0.76)****
F90-F98	**0.69(0.65-0.74)****	**0.74(0.70-0.79)****
Unspecified	0.83(0.78-0.89)	0.58(0.14-2.47)
No axis 1	**0.77(0.69-0.87)****	**0.62(0.55-0.69)****

**p<.05, **p<.01 OR: odds ratio, CI: Confidence intervals Fully adjusted for: age, ethnicity group, sex. Bold: significant results after controlling for multiple comparisons (0.05/30 tests conducted, new p level=0.0017)*

Supplementary Table 2: Unadjusted and fully adjusted logistic regression models for having at least one physical victimisation mention.

Regression type	Unadjusted OR(95% CI)	Fully adjusted OR(95% CI)
**Age**		
0-10 years	**0.47(0.43-0.51)****	**1.19(1.14-1.25)****
11-20 years	0.97(0.92-1.03)	**0.39(0.34-0.42)****
21-30 years	Reference group	
31-40 years	**1.19(1.13-1.26)****	**0.80(0.75-0.84)****
41-50 years	**1.47(1.39-1.56)****	**1.09(1.03-1.15)****
51-60 years	**1.57(1.48-1.66)****	**1.32(1.24-1.41)****
61-70 years	1.10(1.02-1.19)*	1.32(1.24-1.40)*
71-80 years	**0.48(0.44-0.53)****	**0.91(0.84-0.99)****
81-90 years	**0.30(0.27(0.34)****	**0.37(0.34-0.41)****
**Sex**		
Female	Reference group	
Male	**0.72(0.70-0.74)****	**0.69(0.67-0.72)****
**Ethnic group**		
White British (%)	Reference group	
White Other (%)	1.06(0.99-1.13)	1.02(0.96-1.09)
Black (%)	**1.78(1.70-1.86)****	**1.79(1.71-1.88)****
Asian (%)	**1.23(1.14-1.33)****	**1.24(1.14-1.34)****
Other/Mixed (%)	**1.39(1.31-1.48)****	**1.38(1.29-1.47)****
Not Stated (%)	**0.32(0.30-0.33)****	**0.29-0.67-0.72)****
**Diagnostic group**		
F0-F09	**0.27(0.25-0.29)****	**0.34(0.30-0.37)****
F10-F19	**0.88(0.83-0.93)****	**0.60(0.56-0.63)****
F20-F29	**4.37(4.13-4.62)****	**3.36(3.16-3.57)****
F30-F39	**1.55(1.48-1.63)****	**1.39(1.31-1.46)****
F40-F49	1.03(0.99-1.08)	0.96(0.91-1.01)
PTSD	**5.13(4.44-5.94)****	**4.56(3.92-5.32)****
F50-F59	**0.65(0.59-0.72)****	**0.49(0.44-0.54)****
F60-F69	**5.19(4.69-5.75)****	**4.31(3.88-4.80)****
F70-F79	**1.41(1.23-1.63)****	0.99(0.86-1.15)
F80-F89	**0.65(0.61-0.71)****	**0.81(0.74-0.87)****
F90-F98	**0.57(0.55-0.59)****	**0.70(0.67-0.73)****
Unspecified	1.69(0.54- 5.72)	1.16(0.36-4.05)
No axis 1	**0.54(0.50-0.58)****	**0.67(0.618-0.73)****

**p<.05, **p<.01 OR: odds ratio, CI: Confidence intervals Fully adjusted for: age, ethnicity group, sex. Bold: significant results after controlling for multiple comparisons (0.05/56 tests conducted, new p level=0.00089)*

Supplementary Table 3: Unadjusted and fully adjusted logistic regression models for having at least one domestic victimisation mention.

Regression type	Unadjusted OR(95% CI)	Fully adjusted OR(95% CI)
**Age**		
0-10 years	**0.53(0.48-0.57)****	**0.49(0.45-0.54)****
11-20 years	0.99(0.94-1.04)	**0.84(0.79-0.89)****
21-30 years	Reference group	
31-40 years	**1.22(1.16-1.29)****	**1.14(1.08-0.89)****
41-50 years	**1.26(1.19-1.33)****	**1.17(1.10-1.25)****
51-60 years	**1.27(1.20-1.35)****	**1.13(1.06-1.21)****
61-70 years	**0.85(0.78-0.92)****	**0.72(0.66-0.78)****
71-80 years	**0.41(0.37-0.45)****	**0.31(0.28-0.35)****
81-90 years	**0.25(0.22-0.29)****	**0.18(0.16-0.20)****
**Sex**		
Female	Reference group	
Male	**0.47(0.45-0.49)****	**0.44(0.42-0.45)****
**Ethnic group**		
White British (%)	Reference group	
White Other (%)	1.05(0.99-1.12)	1.0390.96-1.10)
Black (%)	**1.37(1.31-1.44)****	**1.39(1.32-1.45)****
Asian (%)	1.08(1.00-1.17)*	1.10(1.02-1.20)*
Other/Mixed (%)	**1.46(1.38-1.55)****	**1.40(1.31-1.49)****
Not Stated (%)	**0.35(0.33-0.36)****	**0.44(0.42-0.45)****
**Diagnostic group**		
F0-F09	**0.23(0.21-0.25)****	**0.33(0.29-0.37)****
F10-F19	**0.82(0.78- 0.87)****	**0.66-0.62-0.70)****
F20-F29	**2.11(2.01-2.22)****	**1.84(1.74-1.95)****
F30-F39	**1.84(1.75-1.93)****	**1.63(1.55-1.72)****
F40-F49	**1.16(1.10-1.21)****	0.98(0.93-1.03)
PTSD	**2.89(2.57-3.27)****	**2.36(2.08-2.69)****
F50-F59	0.85(0.78-0.94)**	**0.53(0.48-0.58)****
F60-F69	**0.85(0.78-0.94)****	**4.03(3.65-4.45)****
F70-F79	0.82(0.71-0.95)**	**0.64(0.54-0.74)****
F80-F89	**0.63(0.58- 0.68)****	**0.77(0.71-0.84)****
F90-F98	**0.65(0.63-0.68)****	**0.78(0.75-0.81)****
Unspecified	1.56(0.49-5.00)	1.01(0.30-3.42)
No axis 1	**0.61(0.57-0.66)****	**0.68(0.62-0.74)****

**p<.05, **p<.01 OR: odds ratio, CI: Confidence intervals Fully adjusted for: age, ethnicity group, sex. Bold: significant results after controlling for multiple comparisons (0.05/83 tests conducted, new p level=0.0006)*

Supplementary Table 4: Unadjusted and fully adjusted logistic regression models for having at least one sexual victimisation mention.

Regression type	Unadjusted OR(95% CI)	Fully adjusted OR(95% CI)
**Age**		
0-10 years	**0.24(0.21-0.28)****	**0.23(0.20-0.27)****
11-20 years	**0.70(0.65-0.74)****	**0.59(0.55-0.63)****
21-30 years	Reference group	
31-40 years	**1.17(1.10-1.25)****	1.10(1.03-1.17)**
41-50 years	**1.33(1.25-1.42)****	**1.26(1.18-1.35)****
51-60 years	**1.32(1.24-1.41)****	**1.18(1.10-1.27)****
61-70 years	0.91(0.83-1.00)*	**0.79(0.72-0.87)****
71-80 years	**0.36(0.31-0.41)****	**0.28(0.24-0.32)****
81-90 years	**0.12(0.09-0.15)****	**0.09(0.07-0.11)****
**Sex**		
Female	Reference group	
Male	**0.45(0.43-0.47)****	**0.42(0.41-0.44)****
**Ethnic group**		
White British (%)	Reference group	
White Other (%)	0.96(0.89-1.04)	0.92(0.85-1.00)*
Black (%)	**1.45(1.38-1.52)****	**1.48(1.41-1.56)****
Asian (%)	0.94(0.86-1.02)	0.93(0.85-1.02)
Other/Mixed (%)	**1.24(1.16-1.32)****	**1.24(1.15-1.32)****
Not Stated (%)	**0.31(0.29-0.33)****	**0.27(0.25-0.29)****
**Diagnostic group**		
F0-F09	**0.16(0.14-0.18)****	**0.24(0.21-0.29)****
F10-F19	**0.78(0.73-0.83)****	**0.58(0.54-0.62)****
F20-F29	**2.72(2.59-2.87)****	**2.25(2.11-2.38)****
F30-F39	**1.53(1.44-1.61)****	**1.22(1.15-1.29)****
F40-F49	1.09(1.03-1.15)**	0.93(0.87-0.98)**
PTSD	**4.09(3.64-4.60)****	**3.64(3.21-4.12)****
F50-F59	0.80(0.71-0.90)**	**0.53(0.47-0.59)****
F60-F69	**5.53(5.08-6.02)****	**4.09(3.74-4.47)****
F70-F79	1.04(0.88-1.22)	0.77(0.65-0.92)**
F80-F89	**0.43(0.38-0.48)****	**0.66(0.59-0.74)****
F90-F98	**0.60(0.57-0.63)****	**0.73(0.70-0.77)****
Unspecified	4.09(3.64-4.60)	1.09(0.28-3.67)
No axis 1	**0.41(0.37-0.45)****	**0.65(0.58-0.73)****

**p<.05, **p<.01 OR: odds ratio, CI: Confidence intervals Fully adjusted for: age, ethnicity group, sex. Bold: significant results after controlling for multiple comparisons (0.05/111 tests conducted, new p level=0.00045).*
